# Imaging predictor of ophthalmic involvement in maxillary sinus cancer during super selective intra-arterial cisplatin infusion and concomitant radiotherapy (RADPLAT)

**DOI:** 10.1186/s13005-021-00285-z

**Published:** 2021-08-16

**Authors:** Hirokazu Ashida, Takao Igarashi, Yosuke Nozawa, Yohei Munetomo, Takahiro Higuchi, Hideomi Yamauchi, Akira Baba, Yukiko Abe, Eiji Shimura, Hisashi Kessoku, Yukio Nishiya, Hiromi Kojima, Hiroya Ojiri

**Affiliations:** 1 Department of Radiology, The Jikei University School of Medicine, 3-25-8, Nishi-Shimbashi, Minato-ku, 105-8461 Tokyo, Japan; 2 Department of Otorhinolaryngology, The Jikei University School of Medicine, 3-25-8, Nishi-Shimbashi, Minato- ku, 105-8461 Tokyo, Japan

**Keywords:** Chemotherapy, Intra-arterial, Maxillary sinus cancer, Radio therapy, Ophthalmic artery, Ethmoid sinus.

## Abstract

**Objective:**

To investigate the predictability of ophthalmic artery involvement in maxillary sinus cancer using preprocedural contrast enhanced CT and MRI.

**Methods:**

We analyzed advanced (T3, T4a, and T4b) primary maxillary sinus squamous cell carcinoma treated with super-selective intra-arterial cisplatin infusion and concomitant radiotherapy (RADPLAT) from Oct 2016 to Mar 2020. Two diagnostic radiologists evaluated the tumor invasion site around the maxillary sinus using preprocedural imaging. These results were compared with the angiographic involvement of the ophthalmic artery using statistical analyses. We also evaluated our RADPLAT quality using complication rate, response to treatment, local progressive free survival (LPFS), and overall survival (OS).

**Results:**

Twenty patients were included in this study. There were ten cases of ophthalmic artery tumor stain and there was a correlation between ophthalmic artery involvement and invasion for ethmoid sinus with statistically significant differences. Other imaging findings were not associated with ophthalmic artery involvement.

**Conclusions:**

Ethmoid sinus invasion on preprocedural imaging could suggest ophthalmic artery involvement in maxillary sinus cancer. It may be useful in predicting prognosis and treatment selection.

## Introduction

Maxillary sinus cancer (MSC) is a rare disease in Japan, representing only about 3 % of all head and neck cancers and 0.5 % of all malignant diseases [1]. Most cases of MSC are at an advanced stage at the initial presentation due to the absence of symptoms at early-stage [2, 3]. However, even if the patient is diagnosed with advanced MSC, lymphatic or hematogenic metastasis is less common [4]. Complete resection is therefore performed for these patients. There are, however, many problems such as impairment of facial function and significant facial deformity after surgical procedures for advanced MSCs [3]. Moreover, there is no indication of surgical resection for patients with stage T4b MSC. Chemoradiotherapy (CRT) is the standard therapeutic option for unresectable MSCs; however, the prognosis of these patients is not satisfactory [5].

Super-selective intra-arterial infusion of high-dose cisplatin with concomitant radiotherapy (RADPLAT) has been performed for patients with locally advanced maxillary sinus squamous cell cancers at several institutions [6]. A previous study showed that the 5-year local progression-free and overall survival rates were 65.8 and 67.9 % for patients with T2 to T4b stage MSCs after the RADPLAT [6]. The presence of internal carotid arterial feeding to the tumor has been reported as a cause of residual tumor or local recurrence and may be one of the problems with RADPLAT [7].

During the interventional radiology (IR) procedure in the RADPLAT approach, in most of the cases, the MSC is fed by the external carotid artery and especially by the third portion of the internal maxillary artery. Other branches of the external carotid artery may feed as the tumor grows. In some cases, it can be fed by the ophthalmic artery. However, it is difficult to infuse the cisplatin due to the risk of ophthalmic complications [8]. Furthermore, some studies reported that intra-orbital tumor infiltration is associated with ophthalmic artery feeding [9, 10]. Iida et al. reported that ante-antral fat pad, orbital, nasal cavity, and ethmoid sinus invasion of MSCs can be the cause of internal carotid feeding, where they analyzed nine cases of intra-arterial-infusion CT angiography (IA-CTA) [7]. An internal carotid angiogram is generally performed to evaluate ophthalmic arterial variation before performing RADPLAT, and ophthalmic artery involvement was often observed in medial invasion of maxillary sinus cancer in our cases. However, to the best of our knowledge, no study has suggested the association of ophthalmic feeding with the site of cancer progression using pre-procedural imaging. Acquisition of the ipsilateral internal carotid angiogram is essential to confirm the anatomical variations in the branching pattern of the ophthalmic artery from the carotid system during IR. However, catheterization into the internal carotid artery can increase the risk of cerebral infarction and should be avoided if possible [11].

We hypothesize that preprocedural CT and MRI can be used to infer whether an ophthalmic artery is involved in the tumor. If the involvement of the ophthalmic artery is suggested before the angiographical procedure, the patient can be informed about the need for embolization for the ophthalmic artery itself, the risk of blindness when embolization is performed to the patient, and the effect of no embolization on treatment outcome. Therefore, we investigated the predictability of ophthalmic artery involvement in maxillary sinus cancer using preprocedural contrast-enhanced CT and MRI.

## Materials and methods

### Patients

This retrospective case-control study was approved by our institutional review board (Approved number; 32–243(10,324)), and the requirement to obtain informed consent for participating in this study from the patients was waived.

Inclusion criteria were as follows:

1. Patients who were pathologically proved primary advanced maxillary sinus squamous cell carcinoma and received RADPLAT from Oct 2016 to March 2020.
2. Contrast-enhanced MRI (CEMRI) and dynamic-contrast-enhanced CT (DCECT) were acquired during 45 days before the RADPLAT.
3. Internal carotid angiography was performed during the first intra-arterial cisplatin infusion procedure.

Exclusion criteria were as follows:

1. Difficult to evaluate preprocedural image due to blurred CEMRI or DCECT.
2. Defect of the ophthalmic artery on the lesion side being recognized on the DCECT or CEMRI.

We divided patients into two groups which were angiographically ophthalmic artery involvement group (n = 10) and non-ophthalmic artery involvement group (n = 10). Each patient’s demographics such as age, sex, BMI, and clinical maxillary sinus cancer stage (8th UICC clinical stage) were described. We also described the duration of the post-treatment follow-up period, treatment completion rate, local progression-free survival (LPFS), overall survival (OS), response to treatment according to Response evaluation criteria in solid tumor guideline, dose limiting toxicities according to Common Terminology Criteria for Adverse Events version 5.0, and whether salvage surgery or neck dissection was performed after the RADPLAT. The presence or absence of tumor stain from ophthalmic artery was considered as the gold standard by two experienced interventional radiologist’s consensus, and statistical analysis was performed to determine the anatomical site that was strongly associated with the tumor stain from the ophthalmic artery.

The inter-observer variability of the preprocedural image evaluation between each reader was analyzed.

## CEMRI acquisition

All patients underwent CEMRI before treatment. MRI examinations were performed on a 3T-MR imaging system (MAGNETOM Skyra; Siemens Healthcare, Forchheim, Germany) using a 20-channel head-neck coil. Before the contrast medium injection, sequences included axial T2-weighted images (TR/TE, 5130/82 ms; slice thickness, 3 mm; flip angle, 180°; 291*448 matrix; scan time, 2 min 5 s), coronal T2-weighted images (TR/TE, 4000/93 ms; slice thickness, 3 mm; flip angle, 150°; 314*448 matrix; scan time, 2 min 6 s) and axial diffusion-weighted images (TR/TE, 6400/60 ms; b value, 50/1000; slice thickness, 3 mm; flip angle, 180°; 211*384 matrix; scan time, 2 min 22 s) in our clinical routine. The FOV ranged from 180 to 210 mm, depending on the patient’s size.

The contrast medium was 0.1 mL/kg gadobutrol (Gadovist, Bayer HealthCare Pharmaceuticals, Wayne, New Jersey) by intravenous hand injection.

Post-contrast-enhanced images were axial T1 weighted images using the same parameters before contrast medium injection and axial volumetric interpolated breath-hold examination (VIBE) (TR/TE, 7 / 3.69 ms; slice thickness 0.9 mm; flip angle, 9°; 256*320 matrix; FOV, 220 mm; scan time, 2 min 48 s). The VIBE images were reformatted to coronal and sagittal images.

### DCECT acquisition

All CT examinations were performed using a 128-slice CT scanner (SOMATOM definition FLASH, Siemens Healthcare, Forchheim, Germany). A non-contrast-enhanced CT scan was performed for the neck, and after injection of the contrast medium, the neck was scanned in the arterial phase, and the area from neck to chest was scanned in the venous phase 100 s after the injection. The contrast medium (100 mL omnipaque 350 - iohexol, GE Healthcare, Bioscience, Piscataway, NJ) was injected using a power injector (DUAL SHOT GX7; Nemotokyorindo, Tokyo, Japan) and injected at a flow rate of 3 ml/s. The scan timing of the arterial phase was determined using the bolus tracking technique. The scan parameters were as follows: 1280.6 mm detector configuration, 1.0 pitch value, 1.0 s per rotation, tube voltage of 120 kV, and effective tube current-time product 165 mAs (using Care Dose4D). Axial images were reconstructed with 1.5-mm thickness without slice gap. Coronal and sagittal images were also reformatted as the same thickness in the arterial and venous phases.

### Angiography

Angiography was performed using a commercial biplane angiography system (Siemens Artis zee biplane, Siemens Healthcare, Forchenheim, Germany). All examinations were performed using standard angiography techniques, including femoral puncture, Seldinger’s technique, 4Fr long sheath placement, and advancing a 4Fr diagnostic catheter through the internal carotid and external carotid artery on the side of the MSC. First, a lateral internal carotid angiogram was acquired to evaluate the ophthalmic artery and choroidal stain to deny retinal feeding from the external carotid artery. Then, the catheter was inserted into the external carotid artery and AP/lateral angiography was acquired. After that, 3D DSA of external carotid angiography was acquired to evaluate the MSC feeders that should be inserted into the microcatheter to infuse cisplatin. The number of feeding arteries such as maxillary artery 3rd portion, transverse facial artery, middle meningeal artery, accessory meningeal artery, facial artery, and ophthalmic artery was described.

In all patients, manual injection of contrast for internal and external carotid angiogram was performed using a 10 mL syringe. The power injection of contrast was performed during 3D DSA acquisition.

### RADPLAT

RADPLAT was performed according to the JCOG1212 (Japan Clinical Oncology Group) protocol, 100 mg/m^2^ of cisplatin administration intraarterially weekly for seven weeks with concomitant 70 Gy / 35 fraction radiotherapy. Cisplatin was injected into all feeding arteries combined with an intravenous infusion of sodium thiosulfate except the ophthalmic artery. The percentage of cisplatin infusion was determined by visual evaluation of Cone Beam CT (CBCT) with contrast medium injected through a microcatheter inserted into each feeding artery. Just after each infusion therapy session, we infused 40 mg of prednisolone sodium succinate into the feeding arteries to prevent angitis resulting in vessel obstruction.

### Image interpretations

#### DCECT and CEMRI

Two diagnostic radiologists with 11 years and 16 years of experience in imaging the head and neck evaluated the presence or absence of MSC invasion into the anatomical sites around the maxillary sinus. They individually analyzed contrast-enhanced head and neck CEMRI referring DCECT using PACS monitor while blind to the clinical information. The definition of the invasion was specified as an obvious extension beyond the bone, and compressive extension was excluded from the definition of the invasion.

These anatomical sites were: (1) ante-antral fat pad, (2) nasal cavity, (3) ethmoid sinus,4) sphenoid sinus, 5) inferior aspect of orbital contents, 6) pterygoid plate, 7) pterygopalatine fossa, 8) masticator muscle, and 9) nasopharynx.

Each of the findings made by the two radiologists were compared to evaluate the reproducibility using interobserver variability with κ-statistics.

After that, the two diagnostic radiologists decided the image finding of each invasion site by consensus and compared to the results of the ophthalmic artery tumor stain.

The inter-observer variability on each invasion site, which was evaluated on CEMRI referring DCECT by the two radiologists, was also evaluated using the к coefficient.

#### Evaluation of ophthalmic artery involvement

The presence or absence of ophthalmic artery involvement of the MSC was evaluated during the procedure in consensus with two interventional radiologists referring to the internal carotid and external angiogram, and the presence of a contrast defect on CBCT of external carotid artery enhancement.

### Statistical analysis

All statistical analyses were performed using SPSS (ver. 25; IBM).

Continuous variables, such as the mean age were compared between the group of patients with and without the ophthalmic tumor stain using the Mann–Whitney U test. Binary variables, such as the presence or absence of each site of tumor invasion, were compared between the two groups using Fisher’s exact test.

OS and LPFS were estimated using the Kaplan–Meier method with differences assessed by using the log-rank test. For all analyses, a p-value < 0.05 was considered statistically significant. For interobserver variability, κ-statistics were used to measure the degree of agreement. Values of 0.81–1.0 were considered to indicate nearly perfect agreement; 0.61.0.80, substantial agreement; 0.41.0.60, moderate agreement; 0.21.0.40, fair agreement; and ≤ 0.20, slight agreement.

## Results

**Fig. 1:**
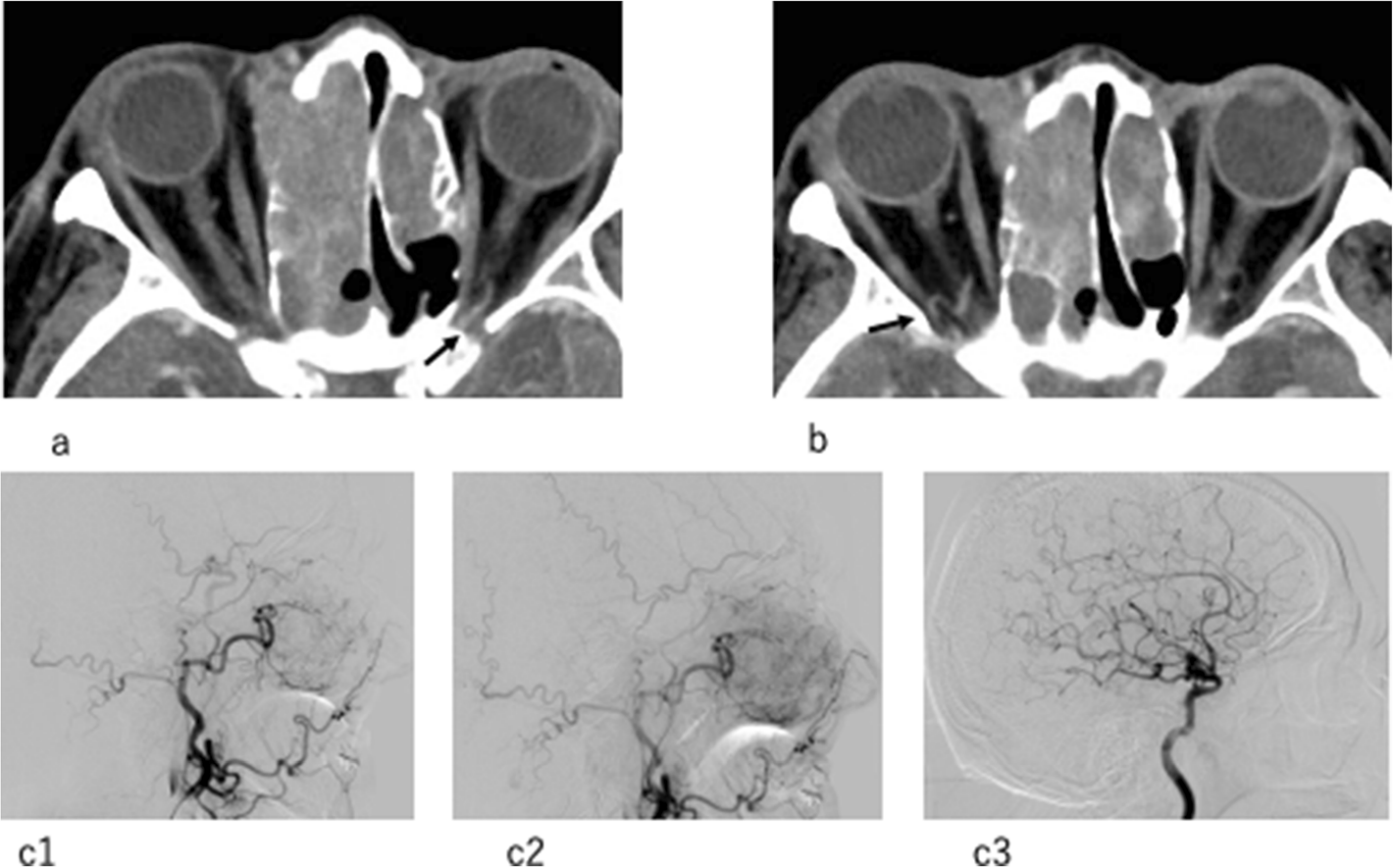
Patient who was excluded because of the absence of an ophthalmic artery on preprocedural DCECT. (**a**) Ophthalmic artery is seen on the left side orbital apex (black arrow). The right ophthalmic artery could not be recognized in the arterial phase of DCECT. (**b**) Instead of that, a tortuous artery (black arrow) running through the superior orbital fissure could be seen. (**c1**, **c2**) The early and delayed phase of external carotid angiogram. The ophthalmic artery connecting from the middle meningeal artery and choroidal crescent could be seen in the delayed phase. (**c3**) There was no ophthalmic artery on the internal carotid angiogram

A total of 21 cases were included in this study. All cases met the inclusion criteria; however, one case was excluded from the registry according to the exclusion criteria because the ophthalmic artery defect was recognized on preprocedural DCECT (Fig. 1). Therefore, 20 cases were eligible for this study.

**Fig. 2:**
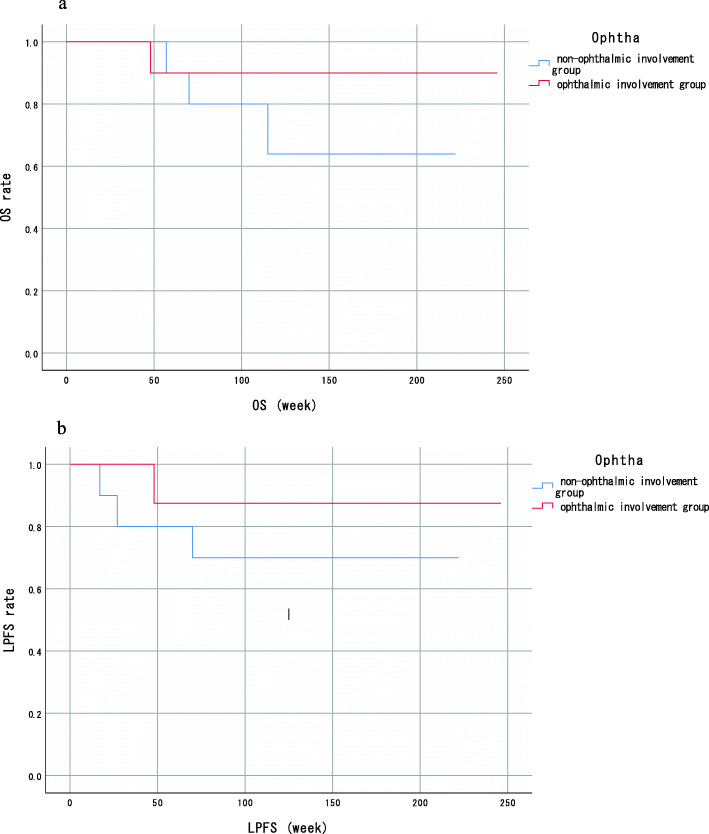
Overall survival. OS curve showing median OS of 173.2 (127.8–218.6 weeks; 95 % CI) weeks in Non-ophthalmic involvement group and 226.2 (189.4–263.0 weeks ; 95 % CI) weeks in Ophthalmic involvement group without significant difference (*p* = 0.35). Local progressive free survival. LPFS curve showing median LPFS of 182.0 (132.4–231.6 weeks; 95 % CI) weeks in Non-ophthalmic involvement group and 203.1 (149.9–256.3 weeks; 95 % CI) weeks in Ophthalmic involvement group without significant difference (*p* = 0.93)

The mean age of all cases was 62.9 (SD: 9.2) years, and 16 male and 4 female patients were included. The patients’ demographics in the presence and absence groups of ophthalmic artery involvement of MSC are shown in Table 1. Regarding the patients’ general background, there was no statistically significant difference between the two groups in age, sex, and BMI. The clinical T stage of the tumors tended to be higher in the group where stain from the ophthalmic artery was observed. Patient outcomes after the RADPLAT such as LPFS and OS are described in Fig. 2 a, b. Mean follow up period including all patients was 128 (98.8–158.3; 95 % CI) weeks and there was no significant difference between ophthalmic artery involvement group and non-ophthalmic artery involvement group in the follow up period 137.2 (82.3-192.1; 95 % CI) weeks vs. 119.9 (84.2-155.6; 95 % CI) weeks, p = 0.82). Regarding treatment completion, all patients were irradiated with 70 Gy. There was no complication related to arterial infusion procedure. However, five patients received reduced infusion times or infusion volume. Among them, one patient received only three weeks of infusions because of suspected anaphylactic reaction for cisplatin; however, it was later determined to be a reaction to antibiotics, and the treatment regime was switched to systemic chemotherapy. Two other patients received six weeks of infusions due to grade 2 renal insufficiency and grade 4 hypokalemia. Two other patients received seven weeks of infusions; however, after the third and fifth infusions, doses were reduced due to grade 3 nausea and grade 2 renal insufficiency. About the treatment response, 70 % of patients were CR and 30 % were PR on contrast-enhanced CT or MRI after RADPLAT. The mean number of feeding arteries including all patients was 2.7 (SD; 0.86). There was significant difference between the two groups (Ophthalmic involvement group was 3.1 (SD; 0.74) and non-ophthalmic involvement group was 2.3 (SD; 0.82). p < 0.05). Regarding additional treatments, two patients received neck dissection for lymph node metastasis and six patients received adjuvant chemotherapy; however, no patient received any other salvage surgery.

**Table. 1:** Patients’ demographics

	Presence of tumor stain from ophthalmic artery *n* = 10	Absence of tumor stain from ophthalmic artery *n* = 10	*p* value
Age (years (SD))	61.4 (7.2)	64.5 (11.0)	0.315
Sex: Male (%)	9/10 (90 %)	7/10 (70 %)	0.582
BMI (Mean (SD));	20.0 (2.35)	21.3 (2.90)	0.426
Clinical stage			
T-factor (T3/4a/4b)	1/3/6	0/7/3	
N-factor (N0/more)	(8/2)	(9/1)	
M-factor (M0/1)	10/0	10/0	

*BMI* Body mass index, *SD* Standard deviation

Imaging findings for each patients described in Table 2 and statistical analyses in Table 3. 50 % (10/20) of cases were recognized as the tumor stain from the ophthalmic artery. There was a significant difference in an invasion into the ethmoid sinus between the groups with and without ophthalmic arterial tumor staining. The sensitivity, positive predictive value, and negative predictive value of the contrast effect from the ophthalmic artery by ethmoid sinus invasion were 100 % (10/10), 91 % (10/11), and 0 % (0/9), respectively. There was no significant difference between the two groups in other invasion sites evaluated using preprocedural images.

**Table. 2:** Preprocedural finding and ophthalmic artery involvement

	Optha on DSA	Ante-antral	Nasal cavity	Ethmoid	Sphenoid	Intaraorbital infra	Pterygoid	PPF	Masticator	Nasopharynx
Case1	○	○	○	○	-	-	○	○	○	○
Case2	○	○	○	○	-	-	○	○	-	○
Case3	○	○	○	○	-	○	-	-	-	-
Case4	-	○	○	-	-	○	○	○	○	-
Case5	○	-	○	○	-	-	-	-	-	-
Case6	○	-	○	○	○	-	○	○	○	○
Case7	-	○	-	-	-	○	○	○	○	-
Case8	-	○	-	-	-	○	-	○	○	-
Case9	-	-	○	-	-	○	-	○	-	-
Case10	-	○	○	-	-	○	○	○	○	-
Case11	-	○	○	○	-	○	-	○	-	-
Case12	○	○	○	○	-	○	-	○	○	-
Case13	-	○	-	-	-	-	○	○	○	-
Case14	-	-	○	-	-	-	-	○	-	-
Case15	-	-	○	×-	-	-	-	-	-	-
Case16	○	○	○	○	-	-	○	-	-	-
Case17	-	○	-	-	-	○	-	-	-	-
Case18	○	-	○	○	-	○	-	○	○	-
Case19	○	○	○	○	-	○	-	-	○	○
Case20	○	○	○	○	-	○	-	-	○	-

*DSA* Digital subtraction angiography; *Ophtha* Ophthalmic; *Ante-antral* Ante-antral fat pad; *Ethmoid* Ethmoid sinus; *Sphenoid* Sphenoid sinus; *Intraorbital infra* Inferior aspect of orbital contents; *Pterygoid* Pterygoid plate; *PPF* Pterygopalatine fossa; *Masticator* Masticator muscle

**Table. 3:** Analysis of preprocedural imaging findings compared with ophthalmic artery involvement

	Presence of tumor stain from ophthalmic artery*n* = 10	Absence of tumor stain from ophthalmic artery*n* = 10	
Invasion site			
Ante-antral space	7/10 (70 %)	7/10 (70 %)	1.000
Nasal cavity	10/10 (100 %)	6/10 (60 %)	0.87
Ethmoid sinus	10/10 (100 %)	1/10 (10 %)	< 0.01
Sphenoid sinus	1/10 (10 %)	0/10 (0 %)	1.000
Inferior orbital	5/10 (50 %)	7/10 (70 %)	0.650
Pterygoid plate	4/10 (40 %)	4/10 (40 %)	1.000
PPF	5/10 (50 %)	8/10 (80 %)	0.350
Masticator muscle	6/10 (60 %)	5/10 (50 %)	1.000
Nasopharynx	4/10 (40 %)	0/10 (0 %)	0.087

*Inferior orbital* Inferior aspect of orbital contents; *PPF* Pterygopalatine fossa

The inter-observer variability of the image findings on each invasion site was as follows:

Ante-antral fat pad, nasal cavity, ethmoid sinus, pterygoid plate, masticator muscle, and nasopharynx were considered as substantial agreement (0.61 < κ < 0.80). The retro-antral fat pad and orbit showed nearly perfect agreement (0.81 < κ < 1.00). Pterygopalatine fossa showed moderate agreement (0.41 < κ < 0.60).

## Discussions

Preprocedural CEMRI with DCECT images of 21 patients with advanced MSC treated with RADPLAT in a retrospective manner were evaluated. DCECT could exclude one patient whose ophthalmic artery was absent. Therefore, 20 cases were then evaluated and the relationship between the tumor invasion site and tumor staining from the ophthalmic artery analyzed. The invasion anatomical sites around the maxillary sinus were selected with referring to the previous literature [7] and the 8th UICC TNM clinical classification.

**Fig. 3:**
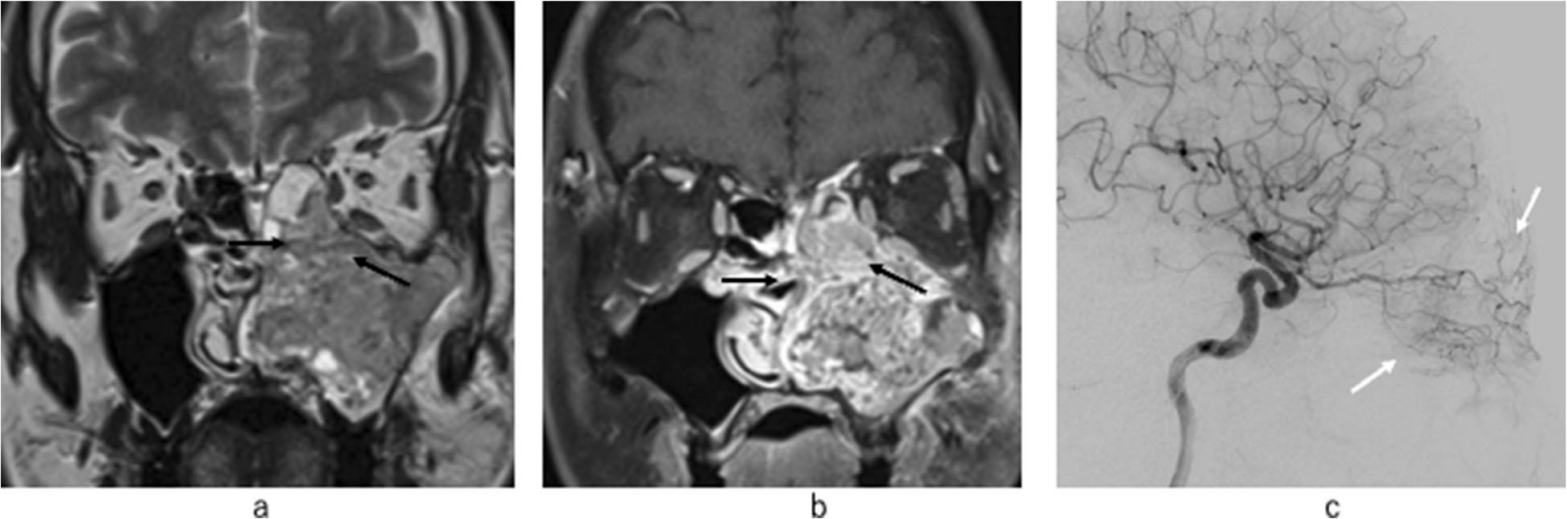
Patient who had left MSC with ethmoid sinus invasion and ophthalmic artery involvement of MSC. (**a**, **b**) Coronal section of T2 weighted image and fat-suppressed contrast enhanced T1weighted image, ethmoid sinus invasion could be recognized on each image (black arrow). (**c**) Lateral view of the internal carotid angiogram showed MSC enhancement (white arrow) from the peripheral branch of the ophthalmic artery

**Fig. 4:**
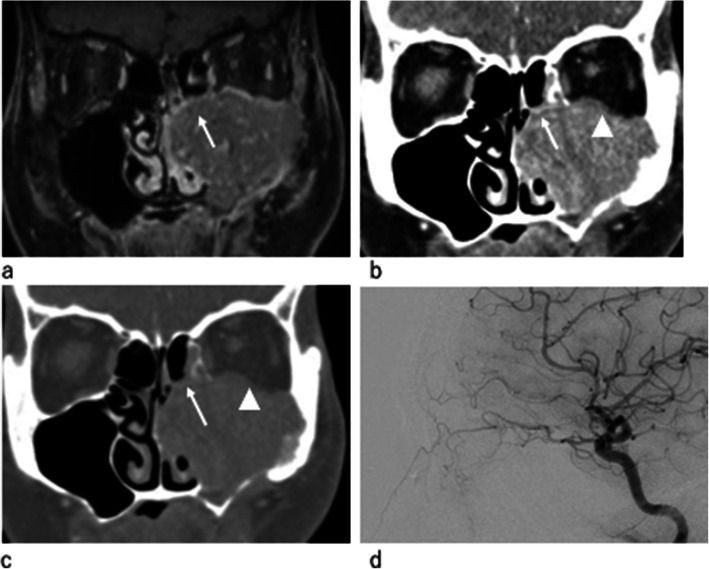
Patient showed swollen extension of MSC in the ethmoid sinus on the coronal section of fat-suppressed T1 weighted image (**a**; white arrow); however, there was no borderline bone destruction on the coronal section of contrast-enhanced CT (**b**, **c**; white arrow), and it was determined that there was no invasion. In this case, it was difficult to determine the presence or absence of invasion into the ethmoid sinus on MRI, and CT was referred to determine the absence of bony destruction. (**b**, **c**; arrow) Infiltration into the orbital floor with bony destruction was noted. (**d**) A lateral view of the left internal carotid angiogram showed no enhancement of MSC from the ophthalmic artery

Regarding the invasion site, a statistically significant positive correlation was found between the ethmoid sinus invasion and ophthalmic arterial tumor stain. From our results, when the ethmoid sinus invasion was present on preoperative images, 91 % (10/11) of the cases had tumor stain from the ophthalmic artery and the internal carotid angiogram was needed to decide whether the ophthalmic arterial chemo infusion or the embolization should be performed (Fig. 3). Cases without ethmoid sinus invasion and internal carotid angiogram might be skipped (Fig. 4).

No other invasion site was statistically correlated with the ophthalmic arterial tumor stain. Iida et al. analyzed IA-CTA in nine patients who underwent RADPLAT and reported that the perfusion territories of the internal carotid artery were seen in the tumor extending to the medial part (nasal cavity and ethmoid sinus), the orbit, and the anterior part of the maxillary sinus [7]. Our results are in agreement for the ethmoid sinus but not for the nasal cavity, orbit, or the anterior part. The reason for this disagreement could be that although the vascularization from the ophthalmic artery can also occur with tumor infiltration into the infraorbital wall [12] or ante-antral fat pad, and nasal cavity, the basic nourishment of these areas are from the inferior orbital artery in the infraorbital wall [12], from the facial artery in the ante-antral fat pad [13], and sphenopalatine artery in the nasal cavity [14]. Supplementary blood flow from the ophthalmic artery can be generated if these vessels are disrupted or hypoplastic, but with the ethmoid sinus. In most of the cases, the nourishment takes place from the anterior or posterior ethmoidal artery, which is the branch of the ophthalmic artery [15].

Furthermore, a nasal invasion is thought to be the pathway for ethmoid sinus extension and is always accompanied by nasal extension in cases of ethmoid sinus extension. In our study, all of the patients who recognized tumor invasion into the ethmoid sinus showed nasal cavity involvement.

Evaluation of the IA-CTA literature also mentioned that filling defects in external arterial cannulated IA-CTA caused internal carotid flow, and filling defects could be one of the factors of local recurrence or residual tumor [7]. Therefore, it is important to be aware of the high risk of a residual or recurrent tumor prior to the treatment to decide on a treatment plan and explain to the patient about the risk of tumor curability and the treatment of the ophthalmic artery.

Our results indicate that internal carotid angiography should be avoided if the MSCs do not show the involvement of the ethmoid sinus. A previous report has shown that diagnostic cerebral angiography causes silent embolism at a relatively high rate. Therefore, they concluded that catheter insertion should be avoided whenever possible, especially for patients with vascular pathology such as atherosclerosis or in cases where insertion was difficult [11]. Because of the overlapping risk factors for head and neck cancer and atherosclerosis and also MSC cases in relatively high age range, there is a high probability that patients with MSCs have a concomitant vascular pathology [16].

The reproducibility of the MSC invasion sites in MRI imaging was generally better than the substantial agreement, and with respect to the Pterygopalatine fossa, the results were in moderate agreement. These results showed that the reproducibility of the invasion site can be considered to be sufficient.

Although the observation period was insufficient, we also examined the prognosis after RADPLAT. The OS and LPFS of our study could not be easily directly compared with the previous study. However, LPFS rate was 80 %, OS rate was 76.2 %, and tumor response rate was 100 % including all patients in our follow up period. Homma et al. reported four weeks high dose (100–120 mg/m^2^ cisplatin) RADPLAT resulted in 83 % of primary disease control rate and 69.3 % of OS [17]. Therefore, our result was comparable with that previous investigation and we thought that our treatment was appropriate. There was no difference with LPFS and OS between ophthalmic involvement group and no ophthalmic involvement group. However, this result has the possibility that significant difference was not detected due to the small sample size. Therefore, further investigation is required to solve these clinical questions.

Our study has several limitations. (1) This was a retrospective, single-institution study, and (2) included a small number of patients. (3) We evaluated the presence or absence of ophthalmic artery involvement of MSCs in consensus by two interventional radiologists during the procedure, mainly referring to the internal carotid angiogram. Therefore, this might lead to bias concerning objectivity and reproducibility.

## Conclusions

The results of this study suggest that preoperative DCECT or CEMRI can predict whether or not the ophthalmic artery is involved in maxillary sinus cancer, and thus may facilitate treatment decisions. In addition, if ophthalmic artery involvement was thought to be negative, internal carotid angiography may be ruled out, suggesting that it may lead to a reduced risk of cerebral infarction.

## Data Availability

The data and materials are available through e-mail from the corresponding author.
